# Blockade of HMGB1 Attenuates Diabetic Nephropathy in Mice

**DOI:** 10.1038/s41598-018-26637-5

**Published:** 2018-05-29

**Authors:** Xiaochen Chen, Jin Ma, Tony Kwan, Elisabeth G. D. Stribos, A. Lianne Messchendorp, Yik W. Loh, Xiaoyu Wang, Moumita Paul, Eithne C. Cunningham, Miriam Habib, Ian E. Alexander, Alexandra F. Sharland, Steven J. Chadban, Huiling Wu

**Affiliations:** 1 Kidney Node Laboratory, Charles Perkins Centre, The University of Sydney, Sydney, NSW Australia; 2 Department of Renal Medicine, Royal Prince Alfred Hospital, Sydney, NSW Australia; 3 Transplantation Immunobiology Group, Sydney Medical School, The University of Sydney, Sydney, NSW Australia; 4 Gene Therapy Research Unit, Children’s Medical Research Institute and The Children’s Hospital at Westmead, Westmead, NSW Australia; 5 Discipline of Child and Adolescent Health, The University of Sydney, Westmead, NSW Australia

## Abstract

Activation of TLR2 or TLR4 by endogenous ligands such as high mobility group box 1 (HMGB1) may mediate inflammation causing diabetic kidney injury. We determined whether blockade of HMGB1 signaling by: (1) supra-physiological production of endogenous secretory Receptor for Advanced Glycation End-products (esRAGE), a receptor for HMGB1; (2) administration of HMGB1 A Box, a specific competitive antagonist, would inhibit development of streptozotocin induced diabetic nephropathy (DN). Wild-type diabetic mice developed albuminuria, glomerular injuries, interstitial fibrosis and renal inflammation. Using an adeno-associated virus vector, systemic over-expression of esRAGE afforded significant protection from all parameters. No protection was achieved by a control vector which expressed human serum albumin. Administration of A Box was similarly protective against development of DN. To determine the mechanism(s) of protection, we found that whilst deficiency of TLR2, TLR4 or RAGE afforded partial protection from development of DN, over-expression of esRAGE provided additional protection in TLR2^−/−^, modest protection against podocyte damage only in TLR4^−/−^ and no protection in RAGE^−/−^ diabetic mice, suggesting the protection provided by esRAGE was primarily through interruption of RAGE and TLR4 pathways. We conclude that strategies to block the interaction between HMGB1 and its receptors may be effective in preventing the development of DN.

## Introduction

Diabetic nephropathy (DN) develops in 30–40% of people with Type 1 or 2 diabetes and consequently has become the most frequent cause of end-stage renal disease^1,2^. New therapeutic strategies are needed to reduce the progression of DN. Evidence from clinical and experimental studies has demonstrated that sterile inflammatory processes triggered by innate immune responses via TLRs and RAGE play vital roles in the pathogenesis and progression of DN^3–7^.

TLRs are innate immune receptors that can be activated by exogenous ligands derived from microbes, and endogenous ligands derived from injury cells^8^. TLR2 and 4 activation by endogenous ligands including high mobility group box 1 (HMGB1), heat-shock proteins (HSPs) and biglycan, leads to translocation of NF-κB^9^ with consequent upregulation of pro-inflammatory cytokines (TNFα & IL6) and chemokines (CCL2), triggering a sterile inflammation as known to participate in the pathogenesis of DN^10–13^. It is well known that RAGE plays a crucial role in the pathogenesis of DN^14^. Similar to TLR2 and 4, engagement of RAGE by HMGB1, can initiate cellular signals that activate NF-κB and trigger pro-inflammatory responses^15^.

Thus, in the context of diabetes, HMGB1 may potentially mediate inflammation by activating any or all of TLR2, 4 or RAGE in DN. Upregulation of TLR4 and HMGB1 expression was evident in the renal tubules of human kidneys with DN^4^. We have found that *in vitro*, high glucose promotes release of endogenous TLR ligands, including HMGB1, by tubular epithelial cells and podocytes, which coupled with upregulation of TLR2 and 4, resulted in activation of NF-κB and consequent production of pro-inflammatory cytokines^5–7^. In support of the *in vitro* findings, we have reported upregulation of TLR2 or 4 and HMGB1 in early diabetic kidneys in STZ-induced diabetes^5–7^. Furthermore we and others have demonstrated that either absence of TLR2 or TLR4 was protective against development of DN in mice^3–6^. Whilst the ligand responsible for TLR activation in DN has not been confirmed, HMGB1 was upregulated in diabetic kidneys and is thus a likely candidate^5,6^.

Endogenous secretory RAGE (esRAGE) is a soluble decoy receptor for RAGE ligands, which serves to bind ligands such as HMGB1 in circulation and prevent their engagement by cell-based receptors^16,17^. Over-expression of esRAGE to generate supra-physiological concentrations in blood therefore has potential to prevent RAGE, but also TLR2 and 4, engagement and activation by soluble ligands such as HMGB1^18,19^.

HMGB1 contains two binding domains, termed the HMGB1 A Box and B Box. The B Box can bind to TLR2, TLR4 and RAGE, leading to NF-κB activation and subsequent inflammatory responses^20^, while A Box alone is a specific competitive antagonist which attenuates HMGB1 induced production of pro-inflammatory cytokines^21^. Treatment with recombinant A Box inhibiting HMGB1 activity is protective in several inflammatory disease models^22–24^.

Whilst activation of TLR2, 4, and RAGE have been shown to contribute to DN, the mechanism(s) of receptor activation in DN has not been confirmed. Targeting interactions between TLRs or RAGE and their shared ligand (HMGB1) may be a clinically relevant strategy to prevent or treat kidney injury but also confirm the mechanism by which TLRs and RAGE are activated in DN. In this study, we utilized two therapeutic strategies to inhibit endogenous HMGB1 activity, by systemic overexpression of esRAGE or administration of recombinant HMGB1 A Box, and determined the impact on the development of experimental DN and the underlying mechanisms.

## Results

### Recombinant adeno-associated virus (rAAV)-mediated expression of esRAGE *in vivo*

**Figure 1:**
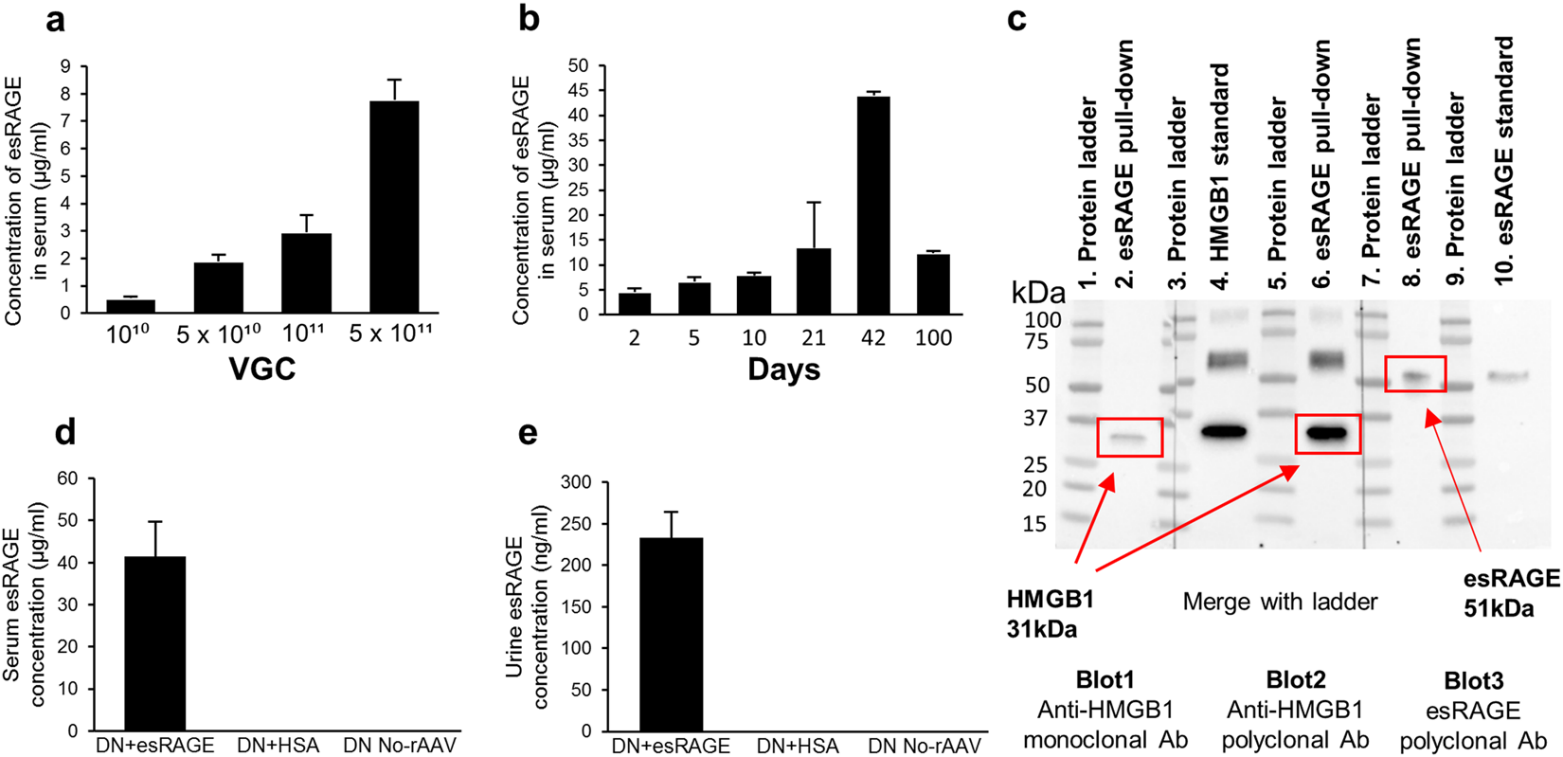
rAAV-mediated expression of esRAGE *in vivo*. (**a**) esRAGE concentration at 10 days post-injection increased in a dose-dependent manner in mice treated with the rAAV-esRAGE vector and was highest in those receiving 5 × 10^11^ VGC (7.8 ± 0.7 µg/ml) (n = 2 per group). (**b**) Timecourse for rAAV-esRAGE at 5 × 10^11^ VGC. The highest expression levels reached at week 6 and remained high at 3 months post-injection (n = 2 per group). (**c**) Confirmation of esRAGE binding to HMGB1 by Co-immunoprecipitation (Co-IP) and Western blot (WB). esRAGE (51 kDa) and rHMGB1 (31 kDa) complex pulled down with anti-RAGE antibody by Co-IP was detected by either anti-HMGB1 or anti-RAGE antibody by WB. Cropped image of blots exposed for 30 seconds, is shown. Multiple exposures of full-length blots are presented in Supplementary Figure S1. (**d**) High levels of esRAGE in serum was detected in DN + esRAGE group four weeks after the injection of rAAV-esRAGE (41.4 ± 8.3 µg/ml, n = 6), while the levels of esRAGE in diabetic mice serum treated with rAAV-HSA (n = 2) or no-rAAV (n = 2) were undetectable. (**e**) esRAGE levels (233.1 ± 31.2 ng/ml) in urine were also detected from these diabetic mice in DN + esRAGE group while esRAGE were undetectable in urine from both diabetic control groups (DN + HSA and DN + No rAAV). Data are presented as mean ± SD.

esRAGE was not detectable in the serum of normal mice, whereas serum esRAGE concentration increased in a dose-dependent manner in mice who received the rAAV-esRAGE vector at 10 days post-injection (Fig. 1a), and was highest in those receiving 5 × 10^11^ vector genome copies (VGC) (7.8 ± 0.7 µg/ml). Mice that received control vector encoding human albumin (rAAV-HSA), 5 × 10^11^ VGC, at 10 days post-injection had a mean serum human albumin concentration of 88 ± 13 µg/ml. Total serum albumin levels in mice were not significantly altered by the vector-mediated expression of human albumin (32.2 ± 1.4 mg/ml, n = 8 over a dose range of albumin vector 1 × 10^10^ VGC, 5 × 10^10^ VGC, 1 × 10^11^ VGC and 5 × 10^11^ VGC, n = 2 per dosage vs 33 ± 2.3 mg/ml, n = 3 normal mice). Intraperitoneal injection of rAAV-esRAGE was well tolerated at all doses; no signs of morbidity were detected following injection, and both alanine aminotransferase levels (27 U/L for rAAV-treated, 33 U/L for controls) and histological appearances in the treated mice were comparable to those in controls, with normal liver morphology and no periportal or lobular inflammatory infiltrates in any animals. Using a dose of 5 × 10^11^ VGC, robust expression of esRAGE was already evident on day 2 post-injection. Expression levels reached a peak at 6 weeks, and remained high at three months post-injection (Fig. 1b). Declining levels seen between 6 weeks and three months are most likely the inevitable consequence of continuing hepatocyte turnover. esRAGE construct binding to HMGB1 was confirmed by co-immuno-precipitation and western blot (Fig. 1c).

Four weeks after the injection of rAAV-HSA or rAAV-esRAGE, high levels of esRAGE were detected in serum from diabetic mice treated with rAAV-esRAGE (DN + esRAGE) but neither in mice treated with rAAV-HSA (DN + HSA) nor in DN + No-rAAV controls (Fig. 1d). esRAGE was also readily detected in urine obtained from DN + esRAGE group mice (Fig. 1e) but was undetectable in urine from both diabetic control groups (DN + HSA and DN + No-rAAV).

### Diabetic mice that received either rAAV-esRAGE or rAAV-HSA developed equivalent degrees of hyperglycaemia

**Figure 2:**
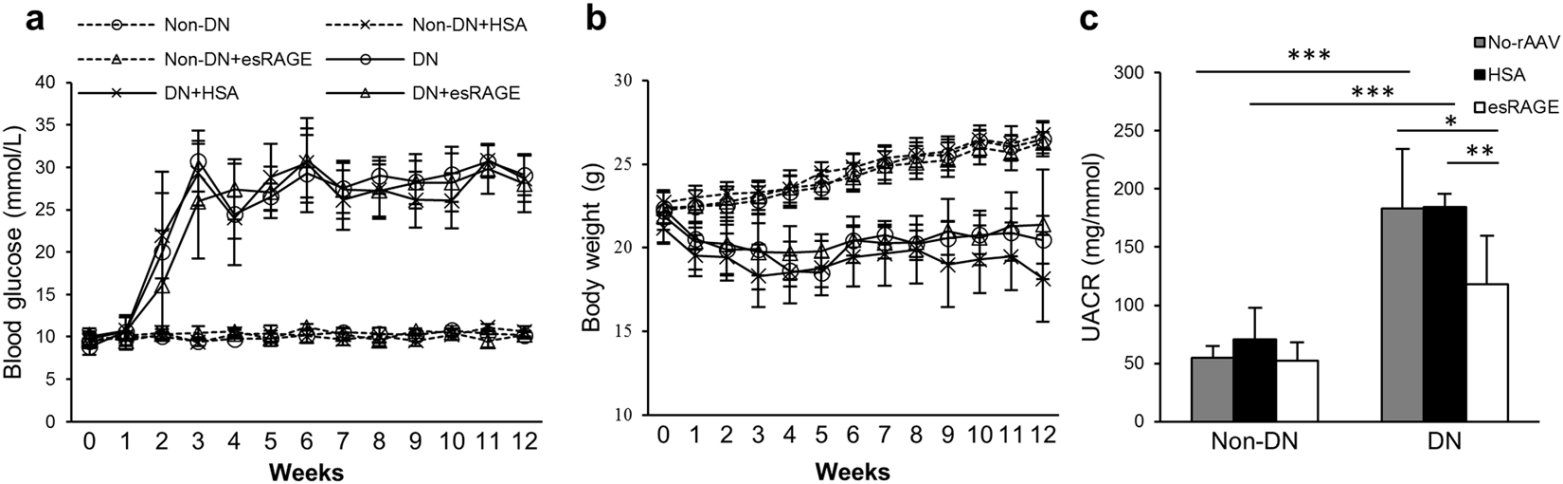
(**a**,**b**) STZ-induced diabetic mice that received rAAV-esRAGE, rAAV-HSA or no virus developed equivalent levels of hyperglycaemia and changes in body weight. (**c**) A significant increase in albuminuria was detected in diabetic mice as compared to controls (UACR for DN:183.4 ± 50.7 and DN + HSA:184.6 ± 9.7 mg/mmol vs Non-DN:55.0 ± 10.0 mg/mmol). rAAV-esRAGE treated diabetic mice, however, had a significantly lower production of albuminuria (UACR: 117.8 ± 41.8 mg/mmol) compared to both diabetic control groups. Data are presented as mean ± SD; *p < 0.05; **p < 0.01; ***p < 0.001.

Diabetic BALB/c mice received rAAV vectors encoding either esRAGE (DN + esRAGE) or HSA (DN + HSA) developed equivalent levels of hyperglycaemia and changes in body weight (Fig. 2a,b) over a 12 week period as did control diabetic mice (DN) that did not receive rAAV vectors.

### Systemic overexpression of esRAGE attenuated albuminuria in DN

12 weeks after the injection of STZ, DN and DN + HSA mice displayed significant albuminuria compared to non-diabetic mice (urine albumin to creatinine ratio (UACR) 183.4 ± 50.7 and 184.6 ± 9.7 mg/mmol vs 55.0 ± 10.0 mg/mmol, *p* < 0.001), while DN + esRAGE mice were partially protected (UACR: 117.8 ± 41.8 mg/mmol) compared to both diabetic control groups (Fig. 2c).

### Overexpression of esRAGE reduced glomerular hypertrophy and injury

**Figure 3:**
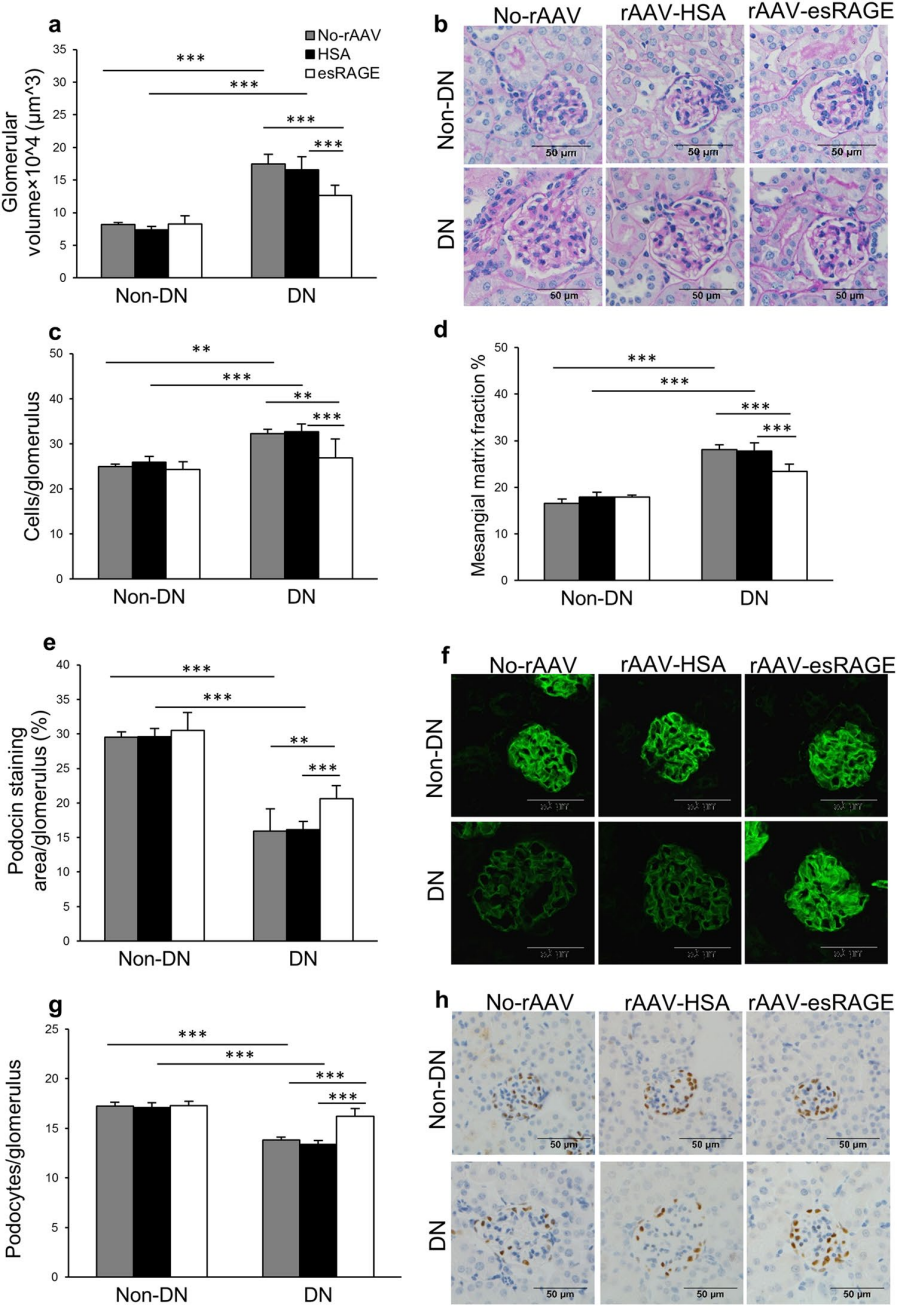
Glomerular injury was reduced in rAAV-esRAGE treated diabetic mice. Both diabetic control mice (no virus and rAAV-HSA) demonstrated significant glomerular injury, including glomerular hypertrophy (**a**,**b**), glomerular hypercellularity (**c**), glomerular mesangial matrix expansion (**d**), podocin injury (**e**,**f**) and WT1^+^ podocyte injury (**g**,**h**) compared to non-diabetic controls. These changes were reduced in rAAV-esRAGE treated diabetic mice. Bars = 50 µm. Data are presented as mean ± SD; **p < 0.01; ***p < 0.001.

DN and DN + HSA kidneys displayed glomerular hypertrophy with a progressive increase in glomerular volume, which was significantly diminished in DN + esRAGE kidneys (Fig. 3a,b). Glomerular hypercellularity was detected in both diabetic control groups, but significantly attenuated in DN + esRAGE kidneys (Fig. 3c). Mesangial matrix expansion, as assessed by computerised morphometric analysis, was evident in both diabetic controls but was also attenuated in DN + esRAGE mice (Fig. 3d).

To evaluate podocyte injury, immunofluorescent staining for podocin and immunostaining for Wilms’ Tumor 1 (WT1) was performed. Depletion of podocin staining was evident in both diabetic control groups as compared to non-diabetic controls, but this reduction was significantly attenuated in DN + esRAGE mice (Fig. 3e,f). Similarly, significant preservation of WT1^+^ podocytes was evident in DN + esRAGE kidneys as compared to DN and DN + HSA groups (Fig. 3g,h).

### Overexpression of esRAGE attenuated interstitial fibrosis and CD68^+^ cell accumulation in diabetic kidney

**Figure 4:**
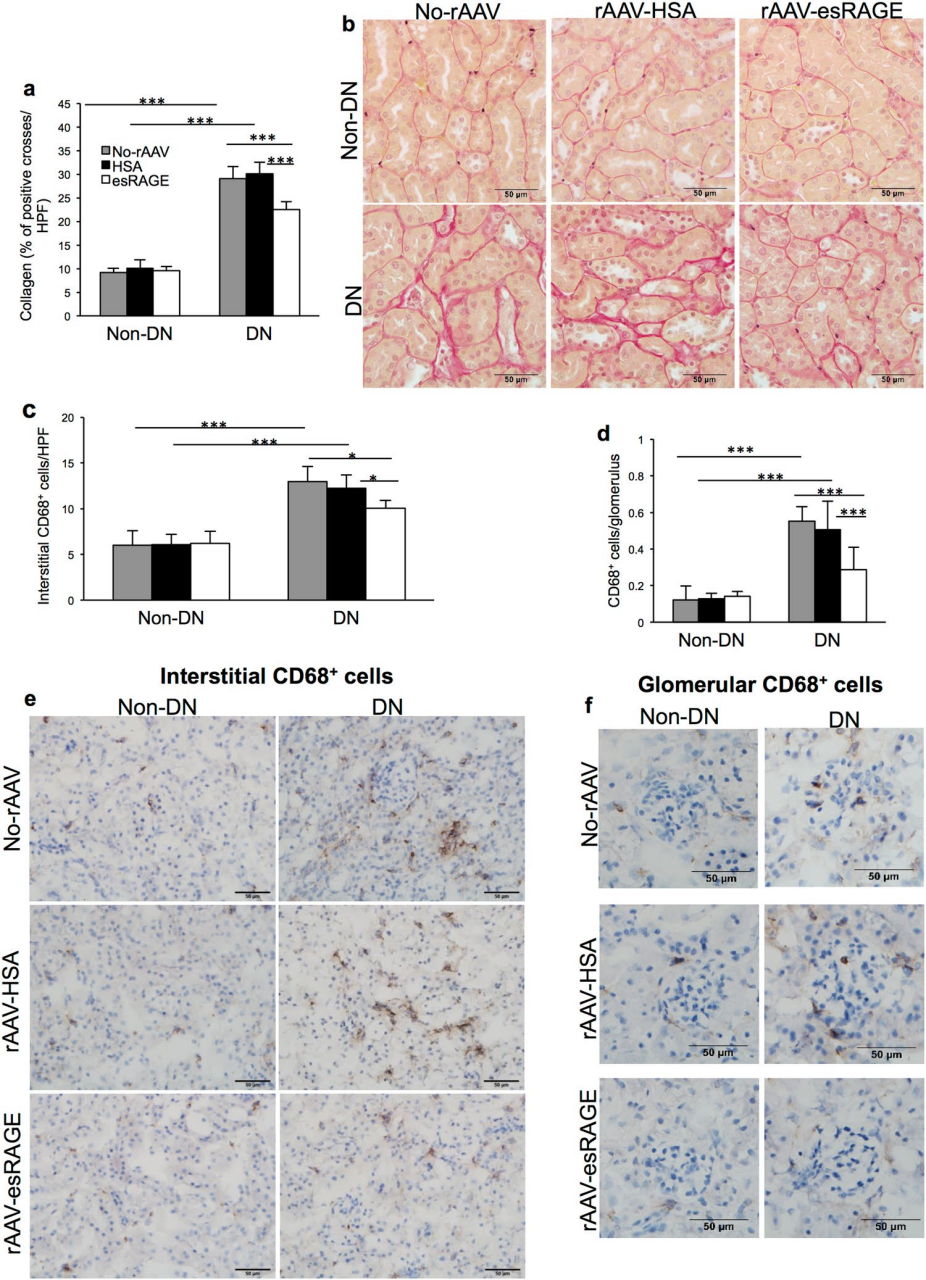
(**a**,**b**) Significant interstitial collagen accumulation was evident in both diabetic control groups (no virus or rAAV-HSA) versus non-diabetic controls, whilst the deposition was significantly attenuated in rAAV-esRAGE treated diabetic mice. **(c**–**f)** Both diabetic control mice (no virus or rAAV-HSA) showed a significant accumulation of CD68^+^ macrophages in both the interstitial (**c**,**e**) and glomerular (**d**,**f**) versus non-diabetic controls, while rAAV-esRAGE treated diabetic mice showed significantly less macrophage accumulation in both the interstitial and glomerular compartments compared to two control diabetic groups. Bars = 50 µm. Data are presented as mean ± SD; *p < 0.05, ***p < 0.001.

Accumulation of extracellular matrix components, including collagen, is a feature of progressive DN^25^. Significant increases in collagen deposition were observed in both diabetic control groups, whilst these changes were attenuated in DN + esRAGE group (Fig. 4a,b). Macrophage accumulation in kidney is an early feature of DN^26^. Accumulation of CD68^+^ macrophages/monocytes in glomeruli and interstitium was significantly increased in diabetic control groups, while esRAGE treated diabetic kidneys (DN + esRAGE) manifested a significantly less accumulation compared to diabetic control groups (Fig. 4c–e).

### Pro-inflammatory molecule expression in DN

**Figure 5:**
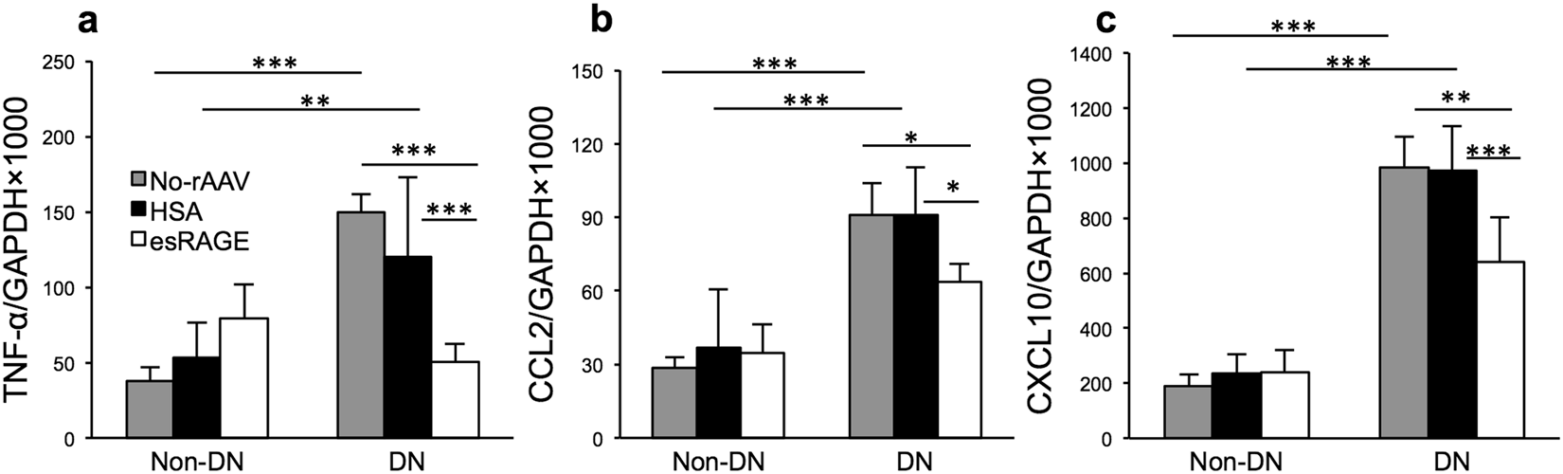
mRNA expression of TLR and RAGE downstream molecules: TNFα (**a**), CCL2 (**b**) and CXCL10 (**c**) were significantly up-regulated in diabetic control groups (no virus or rAAV-HSA) versus non diabetic mice, but were significantly reduced by esRAGE treatment. Results are expressed as a ratio normalised to GAPDH expression. Data are presented as mean ± SD; *p < 0.05, **p < 0.01; ***p < 0.001.

mRNA expression of TLRs and RAGE downstream molecules: cytokine (TNFα), and chemokines (CCL2 and CXCL10) was significantly up-regulated in both diabetic control groups versus non-diabetic mice, whilst esRAGE treated diabetic mice (DN + esRAGE) displayed significantly less up-regulation of these proinflammatory molecules compared to DN and DN + HSA groups (Fig. 5a–c).

### Protective effects of esRAGE involve TLR4 and RAGE signaling

We previously reported that TLR2^−/−^ or TLR4^−/−^ BALB/c mice were partially protected against diabetic kidney injury and that several endogenous ligands, including HMGB1, were upregulated in diabetic kidney^5,6^. TLR2 and 4 are likely activated by endogenous ligands released or expressed within the diabetic milieu. To determine whether the protective effects of esRAGE are attributable to interruption of signaling via the TLR2, TLR4 or RAGE pathways, we studied the effects of rAAV-esRAGE administration to mice deficient in each of these receptors. WT, TLR4^−/−^, TLR2^−/−^ or RAGE^−/−^ mice received an IP injection of 5 × 10^11^ VGC rAAV encoding either esRAGE or HSA two weeks after STZ or vehicle injection. Knock-out and WT mice received STZ injection developed equivalent levels of hyperglycaemia and changes in body weight over a 12-week period.

**Figure 6:**
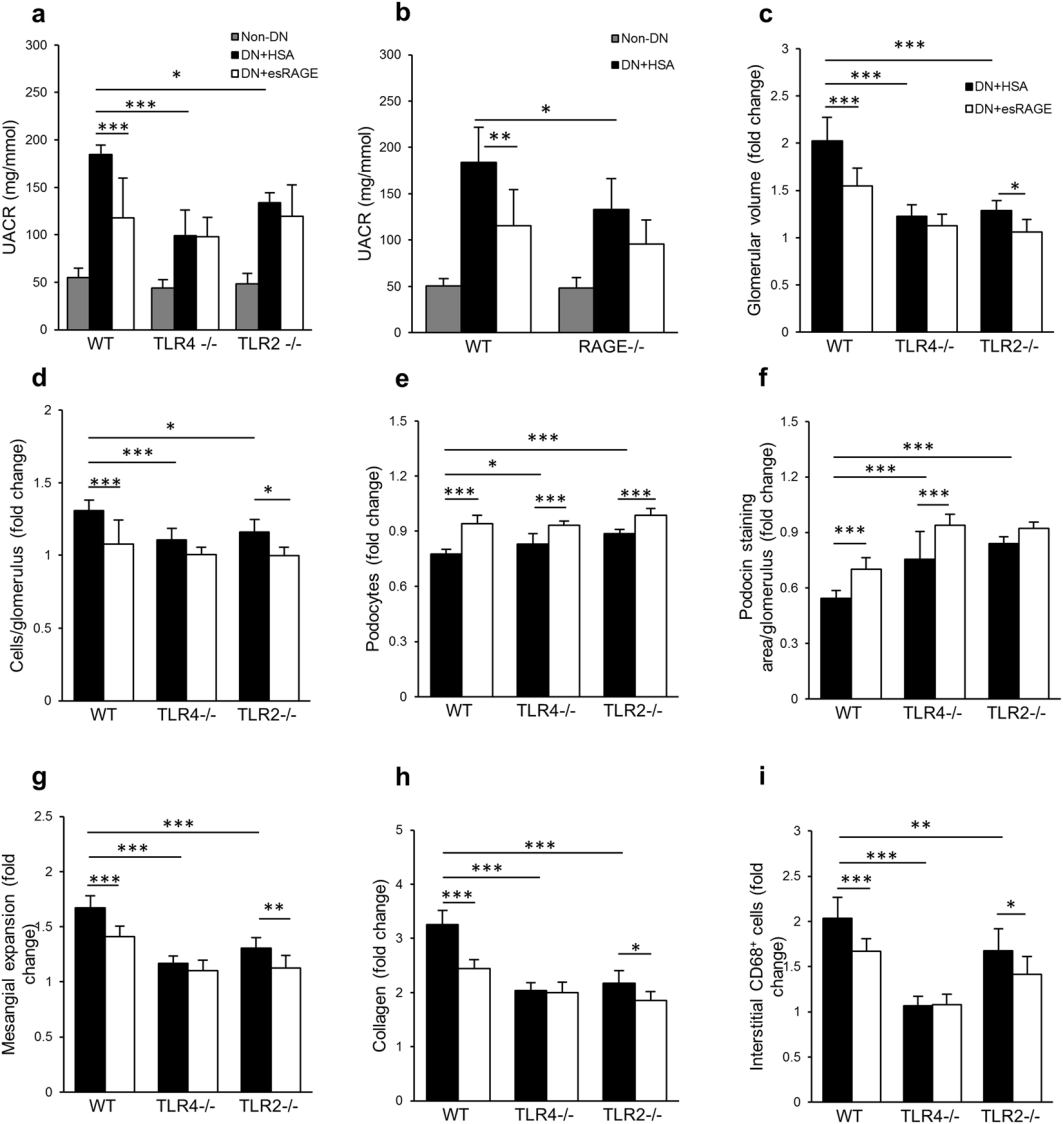
rAAV-esRAGE treated TLR2^−/−^ diabetic kidney displayed a further protection against the progression of DN. (**a**,**b**) TLR2^−/−^, TLR4^−/−^ or RAGE^−/−^ mice treated with either rAAV-HSA or rAAV-esRAGE exhibited less albuminuria compared to WT DN + HSA group, whilst no further protection was observed in diabetic knockout mice treated with esRAGE compared to those treated with HSA. (**c**–**i**) WT diabetic mice treated with rAAV-HSA developed significant renal pathology, including glomerular hypertrophy and hypercellularity, podocyte loss, mesangial expansion, interstitial fibrosis and macrophage accumulation, all of which were attenuated in WT DN + esRAGE mice. TLR2^−/−^ and TLR4^−/−^ diabetic mice treated with rAAV-HSA were partially protected against these diabetic kidney injuries (**c–i**), which is consistent with our previous studies. Treatment with rAAV-esRAGE provided further protection against kidney damage including glomerular hypertrophy (**c**) and hypercellularity (**d**), mesangial cells expansion (**g**), interstitial fibrosis (**h**) and macrophage accumulation (**i**) in TLR2^−/−^ mice, but not in TLR4^−/−^ diabetic mice (**c**,**d**,**g**–**i**). Both TLR2^−/−^ and TLR4^−/−^ diabetic mice treated with rAAV-esRAGE exhibited further protection against podocyte injury (**e**). Additional protection against podocin depletion was evident in rAAV-esRAGE TLR4^−/−^ diabetic mice (**f**). Data are presented as mean ± SD; *p < 0.05, **p < 0.01, ***p < 0.001.

WT diabetic mice treated with rAAV-HSA displayed significant albuminuria at week 12 post-induction of diabetes and this was attenuated in WT mice treated with rAAV-esRAGE (Fig. 6a). TLR2^−/−^, TLR4^−/−^ or RAGE^−/−^ mice treated with rAAV-HSA exhibited less albuminuria with no further protection provided by overexpression of esRAGE (Fig. 6a,b).

**Figure 7:**
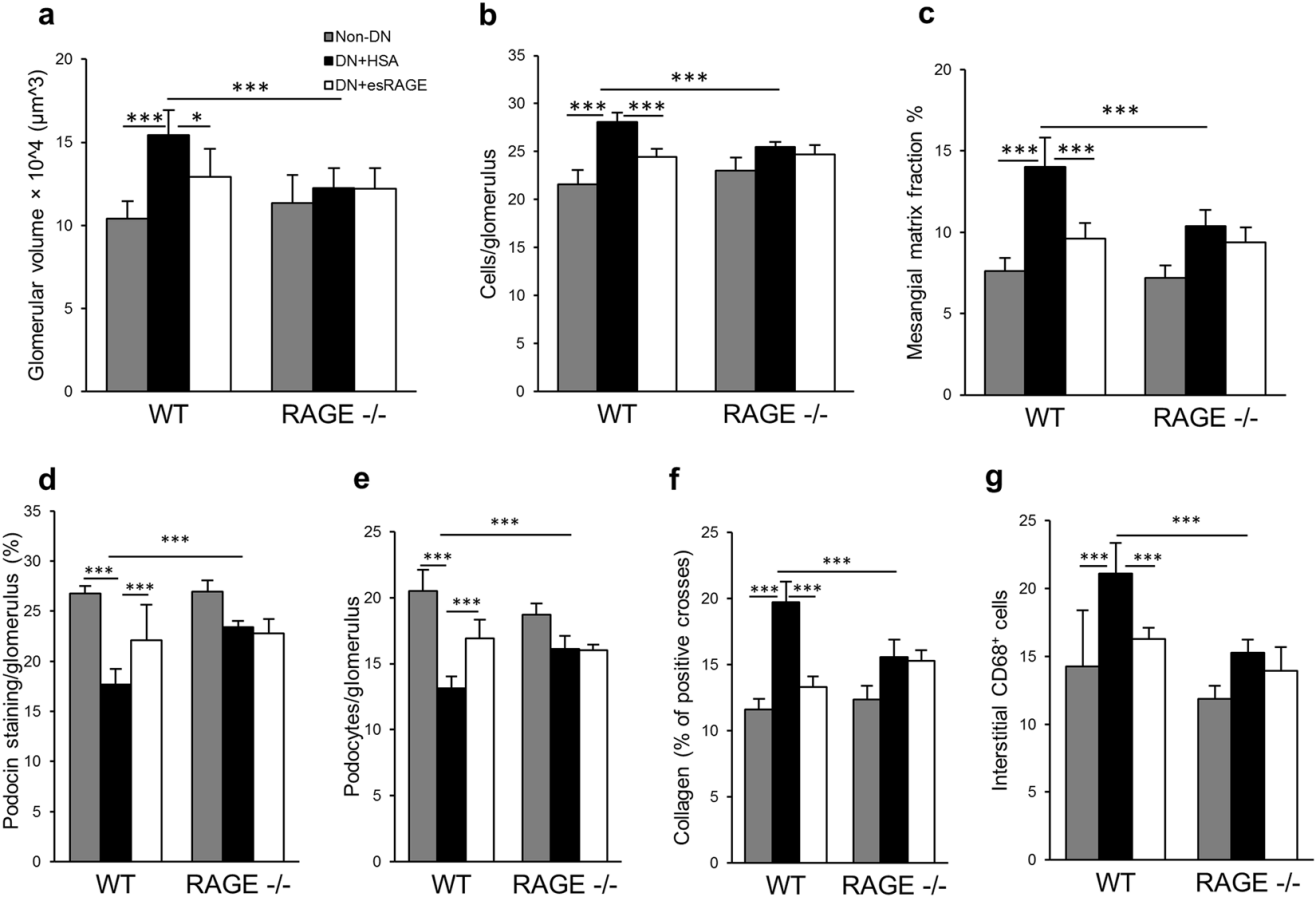
RAGE^−/−^ diabetic mice were partially protected against DN. RAGE^−/−^ diabetic mice received either rAAV-esRAGE or rAAV-HSA treatment were partially protected against DN in terms of glomerular hypertrophy (**a**), hypercellularity (**b**), mesangial cells expansion (**c**), podocin injury (**d**), podocyte injury (**e**), interstitial fibrosis (**f**) and macrophage accumulation (**g**) compared to WT DN + HSA mice. No further protection was observed in RAGE^−/−^ DN + esRAGE mice. Data are presented as mean ± SD; *p < 0.05, ***p < 0.001.

WT BALB/c (Fig. 6c–i) and C57BL/6 (Fig. 7a–g) diabetic mice treated with rAAV-HSA developed significant renal pathology including glomerular hypertrophy and hypercellularity, podocyte loss, mesangial expansion, interstitial fibrosis and macrophage accumulation, all of which were attenuated in WT DN + esRAGE mice. TLR2^−/−^, TLR4^−/−^ diabetic mice treated with rAAV-HSA were also partially protected against these diabetic kidney injuries compared to WT DN + HSA controls (Fig. 6c–i), which is consistent with our previous studies that TLR2^−/−^ or TLR4^−/−^ diabetic mice were protected from the progression of DN^5,6^. We also observed that kidney injuries in RAGE^−/−^ diabetic mice treated with rAAV-HSA were also attenuated compared to WT DN + HSA control group (Fig. 7a–g). Treatment with rAAV-esRAGE provided a modest but significant degree of additional protection against kidney damage including glomerular hypertrophy and hypercellularity, podocyte loss, mesangial cells expansion, interstitial fibrosis and macrophage accumulation in TLR2^−/−^ mice (Fig. 6c–e,g-i), but not in TLR4^−/−^ (Fig. 6c,d,g–i) or RAGE^−/−^ (Fig. 7a–g) diabetic mice. Additional protection against podocin injury was also evident in rAAV-esRAGE treated TLR4^−/−^ diabetic mice (Fig. 6e,f).

### Administration of recombinant HMGB1 A Box attenuates diabetic kidney injury

To confirm that HMGB1 contributes to the development of DN in our model, we administrated recombinant HMGB1 A Box, a specific antagonist of HMGB1 to WT BALB/c mice.

**Figure 8:**
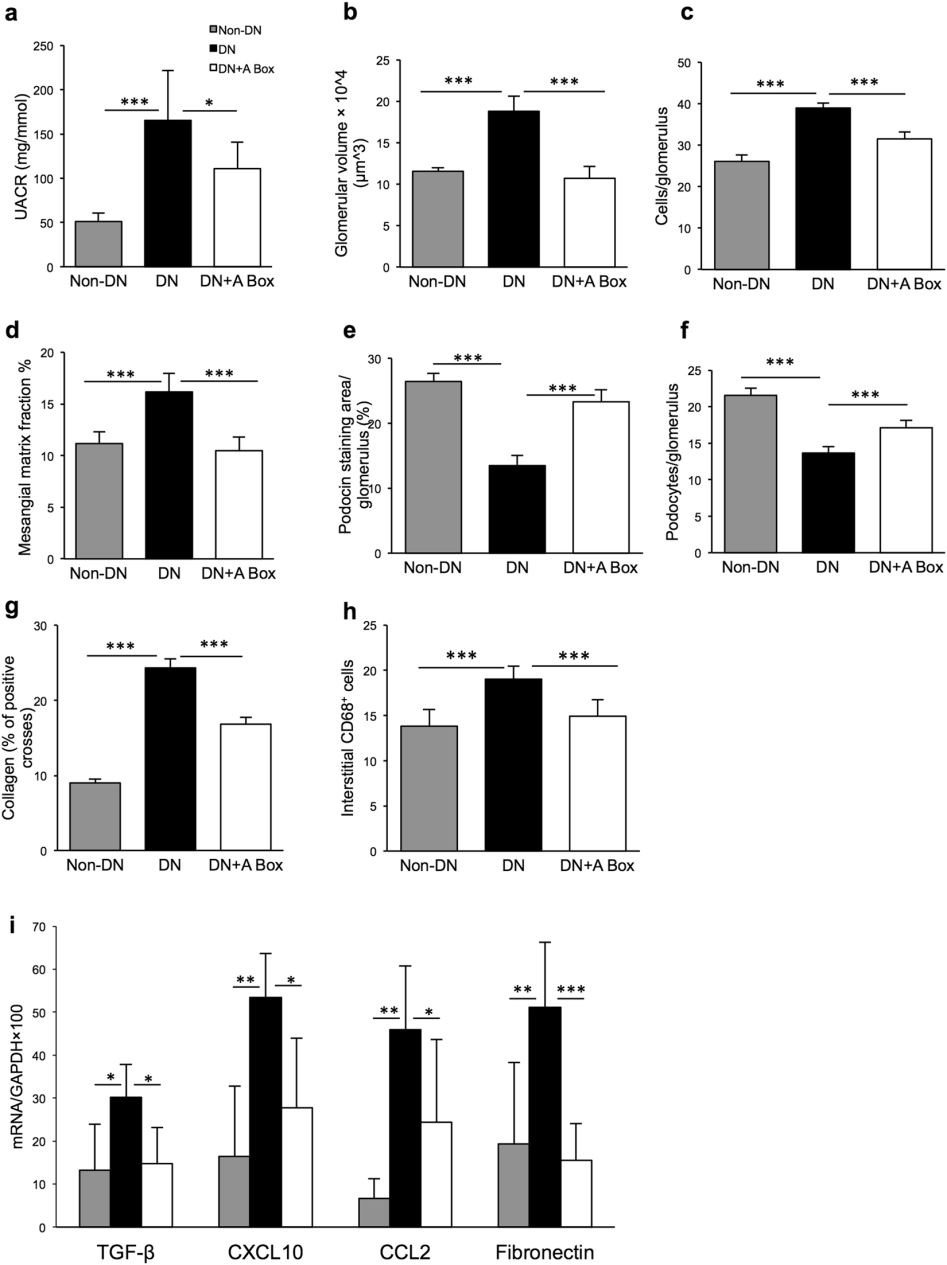
Diabetic mice were protected by the administration of HMGB1 A Box. (**a**) Diabetic mice given saline developed significant albuminuria versus non-diabetic mice (UACR: 165.3 ± 56.3 versus 51.1 ± 9.7 mg/mmol), whilst A Box treated-diabetic mice were protected with significantly less production of albuminuria (110.8 ± 29.6 mg/mmol). (**b**–**h**) WT diabetic mice demonstrated significant glomerular injury including glomerular hypertrophy, glomerular hypercellularity, glomerular mesangial matrix expansion, podocin injury, WT1^+^ podocyte injury, interstitial fibrosis and macrophage accumulation compared to non-diabetic controls, but changes were attenuated by A Box treatment. (**i**) mRNA expression of cytokine (TGF-β and CXCL10), chemokine (CCL2) and pro-fibrotic (fibronectin) genes were significantly up-regulated in saline-treated diabetic kidney versus non-diabetic controls but significantly attenuated in diabetic mice treated with A Box versus saline-treated diabetic mice. Data are presented as mean ± SD; *p < 0.05, **p < 0.01, ***p < 0.001.

WT diabetic mice that received rHMGB1 A Box or saline as controls demonstrated similar patterns of hyperglycaemia and body weight at week 12 post-induction of diabetes. Control diabetic-mice developed significant albuminuria versus non-diabetic mice (UACR: 165.3 ± 56.3 versus 51.1 ± 9.7 mg/mmol, *p* < 0.001), whilst diabetic mice treated with A Box were protected with significantly less albuminuria (110.8 ± 29.6 mg/mmol, *p* < 0.05, Fig. 8a) compared to control diabetic mice.

Saline-treated diabetic-mice developed histological damage including glomerular hypertrophy, glomerular hypercellularity, podocin and podocyte injury, macrophage accumulation and interstitial fibrosis. These changes were significantly attenuated by A Box treatment (*p* < 0.001) (Fig. 8b–h).

mRNA expression of cytokine (TGF-β and CXCL10), chemokine (CCL2) and pro-fibrotic (fibronectin) genes were significantly up-regulated in saline-treated diabetic kidney versus non-diabetic controls but significantly attenuated in diabetic mice treated with A Box versus saline-treated diabetic mice (Fig. 8i). mRNA expression of TNFα was not significantly different between diabetic mice treated with A Box versus saline (data not shown).

## Discussion

In this study, we tested the hypothesis that blocking the interaction between HMGB1 and its receptors would prevent the development of DN. We utilized two novel strategies to block HMGB1 signaling via its receptors, and were thereby able to attenuate development of DN in mice with STZ-induced diabetes, indicating a pathogenic role for HMGB1. The mechanism by which HMGB1 promotes kidney damage in this setting appears to involve inflammation caused by activation of the innate immune system. Activation of the innate immune system by endogenous HMGB1 required TLR4 or RAGE signaling, consistent with previous observations that TLR4^−/−^ mice or RAGE^−/−^ were partially protected against DN. Moreover, the capacity of rHMGB1 A Box to attenuate diabetic nephropathy when given after induction of diabetes, indicates significant therapeutic potential.

Experimental studies have provided compelling evidence that TLRs are actively involved in the development of acute and chronic kidney diseases in a sterile environment. TLR4 and/or 2 are required for the development of kidney damage in response to kidney ischemia reperfusion injury^27,28^, nephrotoxicity^29^ and glomerulonephritis^30,31^. There is increasing clinical and experimental evidence demonstrating that inflammatory processes mediated by innate immunity play a significant role in the pathogenesis of DN. We and others have documented that expression of TLR2, TLR4 and the TLR ligand HMGB1 were upregulated in both human and mouse diabetic kidney, primarily in tubular epithelial cells^5,7^ and that activation of TLR2 and TLR4 contributes to the development of DN^3–5^. Similar to TLRs, aberrant activation of RAGE is involved in the pathogenesis of DN via binding a variety of ligands including HMGB1^14^.

HMGB1 is one of the key endogenous ligands for TLR2, TLR4 and RAGE. Extracellular HMGB1 acts on its target receptors (TLRs and RAGE) leading to nuclear translocation of transcription factors (e.g. NF-κB) and subsequent activation of innate immune responses which have been implicated in mediating several pathological conditions including sepsis^32^, liver^33^ and kidney ischemia reperfusion injury (IRI)^34^, acute lung injury^35^ and diabetes^36^. In experimental kidney IRI, we found that HMGB1 blockade afforded protection from kidney IRI, suggesting that endogenous HMGB1 plays a pathogenic role in kidney IRI^34^. In the current study, we examined whether the blockade of interaction between HMGB1 and its receptors retards the development of DN by inhibiting TLR activation.

Firstly, we used a novel strategy of rAAV-mediated systemic overexpression of esRAGE which could be applied therapeutically. esRAGE is a decoy receptor and can interfere with signalling through RAGE, TLR4 and TLR2 by binding to their shared ligands. Systemic administration of a sufficient quantity of esRAGE can block binding of HMGB1 to both RAGE and TLR2 and 4. Accordingly, deficiency of TLR4, antibody blockade of HMGB1 or administration of 100 µg of recombinant esRAGE daily are all effective in protecting mice against lethal hepatic IRI^37^. In diabetic mice, both the administration of soluble RAGE and adenoviral over-expression of esRAGE have been shown to normalise vascular dysfunction^38,39^. A recent clinical study described an inverse association between esRAGE levels and early kidney injury, suggesting a potential protective role of esRAGE in DN^40^. As human esRAGE is functional in mice, we transduced mouse livers with an AAV vector encoding human esRAGE to achieve systemic overexpression of esRAGE in our model of STZ induced DN. We found that overexpression of esRAGE afforded significant protection against diabetic kidney injury with less albuminuria, glomerular hypertrophy, podocyte injury, macrophage accumulation and interstitial fibrosis. This result suggests that blockade of the interaction between receptors (TLRs and RAGE) and their ligands including HMGB1 attenuated the development of DN.

esRAGE may block HMGB1 signaling via TLR2, TLR4 or RAGE, all of which are well known to contribute to the pathogenesis of DN. To dissect the contribution of each of these pathways in the pathogenesis of DN, we utilised TLR2, TLR4 or RAGE knockout mice to examine whether the protective effects of systemic overexpression of esRAGE would be negated by the absence of individual receptors. Whilst we confirmed previous reports that all three pathways are required for full development of STZ induced DN, the overexpression of esRAGE provided further protection to TLR2 deficient mice, but not TLR4 or RAGE deficient mice. This suggests the protective effect of esRAGE in DN is predominantly mediated via interruption of ligand activation of TLR4 and RAGE, the likely ligand being HMGB1. We observed that esRAGE provided additional protection of podocytes in TLR4^−/−^ mice, implying that a protective effect on podocytes provided by esRAGE may operate through blocking the RAGE pathway, rather than TLR2 or 4. The protective effect of esRAGE may vary by cell type, due to differences in cell-specific pathways of damage in DN. esRAGE provided no additional protection to the RAGE^−/−^ mice, suggesting some degree of redundancy between TLR4 and RAGE in mediating DN.

To confirm that HMGB1 plays a pathogenic role in DN, we used an efficient and potentially clinically relevant strategy of rHMGB1 A Box administration which competitively antagonizes HMGB1 binding to TLRs and RAGE. This strategy appears to be highly specific for HMGB1, as indicated by *in vitro* work demonstrating that HMGB1 A Box can inhibit HMGB1-induced TNF and IL-1β release, but not IL-1β-induced TNF release^21^. Administration of A Box has been shown to afford protection in experimental models of sepsis^21^, endotoxin (LPS)-induced acute lung injury^22^, myocardial infarction^23^ and inflammatory arthritis^24^. Treatment of allograft recipients with A-box significantly prolonged cardiac allograft survival, which was associated with reduced allograft pro-inflammatory cytokine expression of TNFα and IFNγ, and an impaired Th1 immune response^41^. Consistent with these experimental data, we found that A Box provided significant protection against the development of DN. A Box clearly has therapeutic potential for the clinic.

In summary, expression of supra-physiological amounts of esRAGE via administration of rAAV-esRAGE, and treatment with recombinant HMGB1 A Box were successful in attenuating kidney inflammation and damage in a murine model of DN. Effects were likely mediated by TLR4 and RAGE pathways. Treatment with HMGB1 A Box to specifically inhibit HMGB1 activity was protective in DN, confirming a pathogenic role for HMGB1 in DN. Our two novel strategies to block HMGB1 signaling via its receptors indicate significant therapeutic potential.

## Methods

### Animals

WT BALB/c and C57BL/6 mice were obtained from the Animal Resource Centre (Perth, Australia). TLR2^−/−^ and TLR4^−/−^ mice on BALB/c background were provided by Animal Service of Australian National University with permission from Professor S Akira (Osaka University, Osaka, Japan). RAGE^−/−^ mice on C57BL/6 background were provided by Prof. Forbes (The University of Queensland, Australia). The mice were bred and housed in a specific pathogen-free facility at the University of Sydney. Male mice aged 8–9 weeks were used in experiments. All animal experiments were performed with the approval of the animal ethics committee of the University of Sydney. The methods were carried out in accordance with the approved guidelines and regulations.

### Induction of diabetes

Mice were fasted for 4 hours and then injected with 55 mg/kg intraperitoneal (IP) streptozotocin (STZ) or vehicle for 5 consecutive days. Mice with a blood glucose level over 16 mmol/L were considered to have developed diabetes. Mice were killed at week 12 post-induction of diabetes.

### Generation of liver-specific rAAV encoding either esRAGE or HSA

A cDNA sequence encoding human esRAGE (Genbank accession number AB061668) or serum albumin (HSA) was synthesised by GeneArt AG (Regensburg, Germany). The esRAGE cDNA was subcloned into the pAM2AA plasmid backbone incorporating the liver-specific human a-1 antitrypsin promoter and human ApoE enhancer flanked by AAV2 inverted terminal repeats to produce the novel vector pAM2AA-esRAGE or pAM2AA-HSA^42^. After plasmid purification and sequence confirmation, pAM2AA-esRAGE or pAM2AA-HSA was used as the expression vector in recombinant adeno-associated virus 2/8 (rAAV2/8) vector production. The vector (rAAV2/8) was packaged by triple transfection in human embryonic kidney (HEK293) cells with three plasmids: pAM2AA-esRAGE or pAM2AA-HSA, pXX6 (helper plasmid) and p5E18VD2/8 (encapsulating plasmid). rAAV virions were purified by ammonium sulphate precipitation and ultracentrifugation on a caesium chloride gradient, followed by dialysis and concentration. The virion titer was quantified by quantitative real time PCR as previously described^43^.

### Confirmation of binding of esRAGE to HMGB1 by co-immunoprecipitation and western blot

HEK293D cells were cultured and transfected using Lipofectamine 2000 (Thermo Fisher Scientific). The supernatant was collected and esRAGE in the supernatant was confirmed by ELISA (B-Bridge International K1009-1) and Western blot. The supernatant containing esRAGE was mixed in equal parts (100 μl) with recombinant HMGB1 (0.1 mg/ml, Sigma H4652) and incubated for 1.5 hours at 37 °C. Using Pierce Co-immunoprecipitation Kit (Thermo Fisher 26149), the esRAGE-HMGB1 complex is then pulled down with anti-RAGE (Abcam ab37647) antibody coupled resins.

The co-immunoprecipitated proteins were then separated by SDS-PAGE and transferred to nitrocellulose membranes (Bio-RAD). The membranes were blocked and then incubated either with anti-HMGB1 (Abnova H00003146-M08 or Abcam ab18256) or anti-RAGE (Abcam ab37647) antibodies overnight, washed and incubated with horseradish peroxidase-conjugated antibody. Bands were visualized by chemiDoc MP imaging system using enhanced chemiluminescence (Amersham Biosciences, Piscataway, NJ).

### Confirmation of rAAV mediated esRAGE expression *in vivo*

C57BL/6 mice received IP injections of rAAV encoding esRAGE at doses ranging from 10^10^ to 5 × 10^11^ vector genomes copies (VGC)/mouse. At day 10 post-injection, mice were sacrificed. Serum and liver were collected for assessment of esRAGE protein expression. Serum and urine esRAGE was quantitated using a sandwich ELISA, specific for human esRAGE (B-Bridge International K1009-1).

### Experimental Design

At two weeks after STZ or citrate buffer injection, both diabetic (DN) and non-diabetic (Non-DN) mice received either an IP injection of 5 × 10^11^ VGC rAAV encoding esRAGE or 5 × 10^11^ VGC rAAV encoding HSA (control vector) or no vector treatment in the following groups of Balb/c mice: (1) DN + esRAGE n = 10; (2) DN + HSA n = 7; (3) DN n = 4; (4) Non-DN + esRAGE n = 5; (5) Non-DN + HSA n = 5; (6) Non-DN n = 5. (7) TLR2^−/−^ DN + esRAGE n = 7; (8) TLR2^−/−^ DN + HSA n = 6; (9) TLR2^−/−^ Non-DN n = 4; (10) TLR4^−/−^ DN-esRAGE n = 8; (11) TLR4^−/−^ DN + HSA n = 7; (12) TLR4^−/−^ Non-DN n = 6, and C57BL/6 mice: (1) RAGE^−/−^ DN + esRAGE = 10; (2) RAGE^−/−^ DN + HSA = 9; (3) RAGE^−/−^ Non-DN n = 5; (4) DN + esRAGE n = 8; (5) DN + HSA n = 8; (6) Non-DN n = 5.

At two weeks after STZ injection, BALB/c mice received an IP injection of recombinant HMGB1 A Box (HMGBiotech) 400 µg/animal or saline, three times a week for 10 weeks in the following groups: (1) DN n = 6, (2) DN + A Box n = 9, (3) Non-DN, n = 5.

### Sample Collection

Urine, kidney, spleen and pancreas were collected at sacrifice at week 12 post-induction of diabetes. Tissue slices were fixed with 10% neutral-buffered formalin for paraffin embedding, frozen in OCT compound or snap frozen in liquid nitrogen for mRNA extraction.

### Quantifications of albuminuria and urine creatinine

Mouse urine albumin was quantified using the Mouse Albumin ELISA Quantitation Set (Bethyl Laboratories, Montgomery, TX, USA) as described previously^5,6^. BD Falcon ELISA plates (BD Biosciences) were coated with a goat anti-mouse albumin antibody, then rinsed with washing buffer and blocked with assay diluent. Diluted urine samples were applied in triplicate to the plate, along with a reference mouse serum albumin standard dilution series, and incubated for 1 hour at room temperature. The plate was rinsed and incubated with HRP conjugated mouse albumin antibody for 1 hour. Once washed, the plate was incubated with substrate solution for 10 minutes before adding the stop solution. Urine albumin concentration was analysed by microplate reader software (BMG Labtech).

Urine creatinine was measured enzymatically by the Biochemistry Department of Royal Prince Alfred Hospital, Sydney, Australia.

### Histology

Periodic acid–Schiff (PAS) and Picro-Sirius red (PSR) staining were performed on 3 μm and 5 μm formalin-fixed kidney sections, respectively. Total glomerular cellularity was determined by tallying nuclei in glomerular cross-sections using Image Pro Premier 9.0. Glomerular tuft area (A_G_) was measured under microscopy using DP2-BSW software V2.2, OLYMPUS. Mean glomerular volume (V_G_) was calculated using the formula described by Weibel and Gomez^44^; V_G_ = (β/κ) × (A_G_) ^3/2^, where κ = 1.1 (size distribution coefficient) and β = 1.38 (shape coefficient for spheres). Glomerular volume was measured in 20 hilar glomerular tuft cross-sections per animal^45,46^. In each glomerular tuft, mesangial area was defined as positive staining with PAS and enumerated by image analysis software (Image Pro Premier 9.0), expressed as a percentage of total glomerular area. Interstitial collagen on PSR-stained sections was assessed by point counting using an ocular grid as described by McWhinnie *et al*.^47^ in at least 20 consecutive fields (×400 magnification) and expressed as expressed percentage (%) of positive grid points (crosses) per high power field (HPF). Only interstitial collagen was counted, and vessels and glomeruli were excluded.

### Immunostaining and Quantification

Immunohistochemistry staining for CD68 was performed on frozen sections while WT1 protein detection was performed on formalin-fixed sections as previously described^5,6^. Sections were then incubated with horse serum followed by incubation with primary antibodies: rat anti-mouse CD68 antibody (ABD Serotec Inc., Oxford, UK) or rabbit anti-WT1 antibody (Abcam, Cambridge, UK). Endogenous peroxidase activity was blocked and then incubated with biotinylated anti-rat IgG or anti-rabbit IgG (BD Biosciences, Pharmingen). A Vectastain ABC kit (Vector Laboratories Inc) was applied to the tissue followed by DAB solution (DAKO) according to the manufacturer’s instructions.

Immunofluorescent staining for podocin was performed on 7 μm acetone-fixed frozen sections. After blocking with 10% normal horse serum, sections were incubated with a rabbit anti-NPHS2 antibody (Abcam) at 4 °C overnight. For detection, sections were incubated with an Alexa Fluor 488-conjugated anti-rabbit antibody for 1 hour at room temperature in the dark.

Analysis of interstitial CD68^+^ cells was performed by assessing 20 consecutive high-power fields (HPFs × 400 magnification) of the cortex in each section. Using an ocular grid, the number of cells stained positive for each antibody was counted and expressed as cells per field. WT1^+^ cells were counted in glomerular-cross sections and output expressed as podocytes per glomerular.

Analysis of podocin staining was performed on cross section images that were recorded at the same time of 0.01 second exposure time. All exposure settings were kept constant for each sample. The glomerular area expressing podocin was assessed in glomerular cross-sections using Image-Pro and expressed as the percentage of positive staining of glomerular areas and output expressed as percentage of podocin staining area per glomerular. The threshold for positivity was determined by the highest background fluorescence in the non-glomerular area for each section.

### Real-time PCR

Total RNA was extracted using TRIzol (Invitrogen). cDNA was synthesised using Oligo d(T)_16_ primers (Applied Biosystems, Foster City, CA) and the SuperScript III reverse transcriptase kit (Invitrogen) according to the manufacturer’s instructions. cDNA was amplified in Universal Master Mix (Applied Biosystems) with gene-specific primers and probes, using the Rotor-Gene 6000 (Corbett Life Science). Specific TaqMan primers and probes for TNF-α, CCL2, CXCL10, TGF-β1, fibronectin and GAPDH were used as previously described^27^. All of the results are expressed as a ratio normalised to GAPDH expression.

### Statistical analysis

All data are presented as mean ± SD. Multiple groups were compared using one- or two-way ANOVA with *post-hoc* Bonferroni’s correction (GraphPad Prism 6.0 software). A *p* value less than 0.05 was considered statistically significant.

### Data availability

All data generated during and/or analysed during the current study are available from the corresponding author upon reasonable request.

## Electronic supplementary material

Supplementary information
