# Supplementary material for: Blockade of HMGB1 Attenuates Diabetic Nephropathy in Mice
Source: Sci Rep. 2018 May 29;8:8319. doi: 10.1038/s41598-018-26637-5 (PMC5974129; doi:10.1038/s41598-018-26637-5)
Supplement: Supplementary file 1 — Supplementary information [file 41598_2018_26637_MOESM1_ESM.pdf]

Blockade of HMGB1 Attenuates Diabetic Nephropathy in Mice

Xiaochen Chen[1], Jin Ma[1], Tony Kwan[1], Elisabeth G.D. Stribos[1], A.Lianne Messchendorp[1], Yik W. Loh[1], Xiaoyu Wang[1], Moumita Paul[3], Eithne C. Cunningham[3], Miriam Habib[3], Ian E. Alexander[4, 5], Alexandra F. Sharland[3], Steven J. Chadban[1, 2] and Huiling Wu[1, 2]

[1]Kidney Node Laboratory, Charles Perkins Centre, The University of Sydney,

[2]Department of Renal Medicine, Royal Prince Alfred Hospital, NSW, Australia,

[3]Transplantation Immunobiology Group, Sydney Medical School, The University of Sydney,

[4]Gene Therapy Research Unit, Children's Medical Research Institute and The Children's Hospital at Westmead, Westmead, NSW, Australia,

[5]Discipline of Child and Adolescent Health, The University of Sydney, Westmead, NSW, Australia

Corresponding author:

Associate Professor Huiling Wu

Kidney Node Laboratory,

Room 5117, Charles Perkins Centre D17

The University of Sydney, New South Wales 2006, AUSTRALIA

Email : huiling.wu@sydney.edu.au

Supplementary Figure

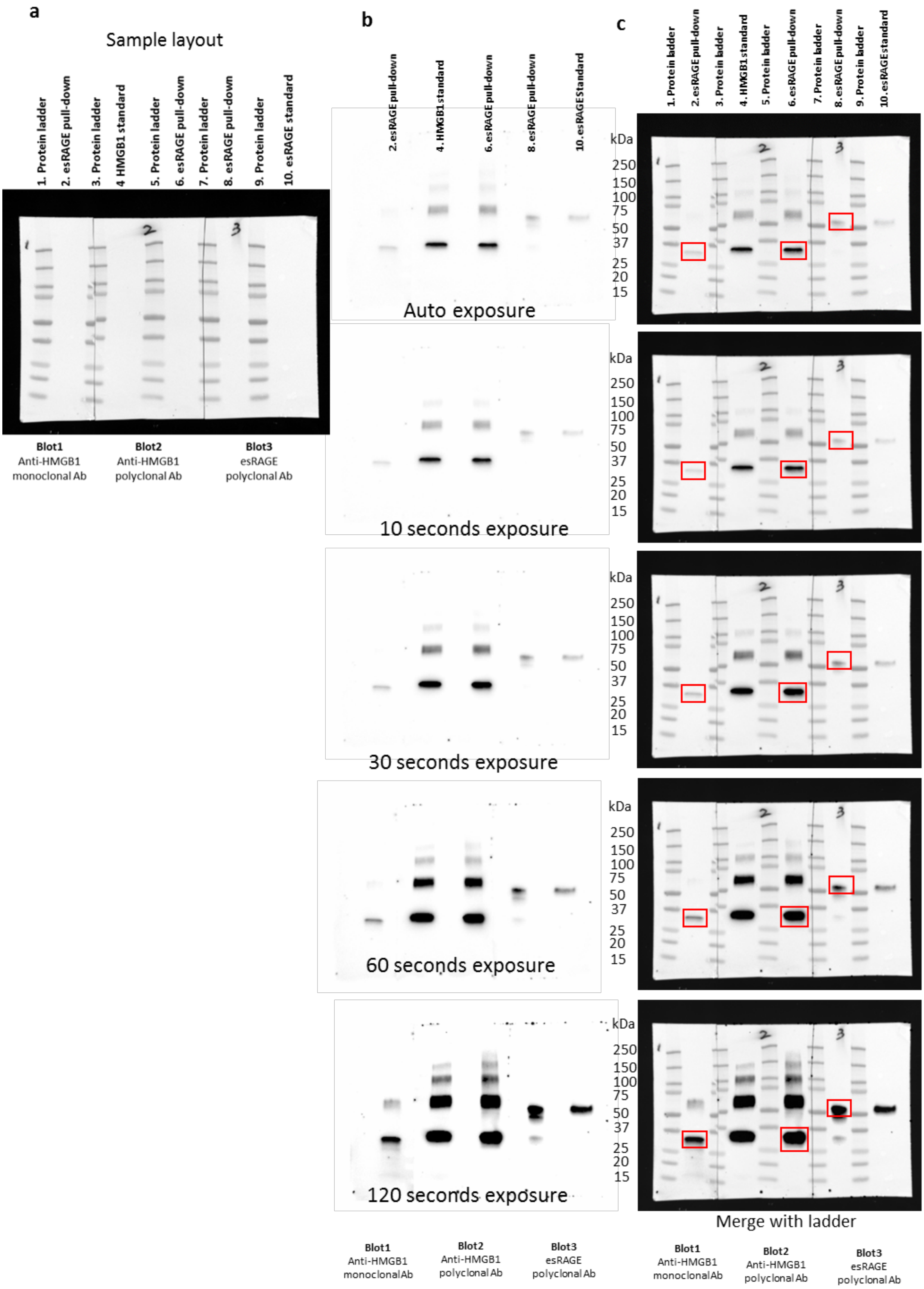

Figure S1. esRAGE construct binds HMGB1. esRAGE (51kDa) and rHMGB1 (31kDa) complex pulled down with anti-RAGE antibody by Co-IP was detected by WB with either anti-HMGB1 or anti-RAGE antibody. (a) Sample layout for Western blot showing protein ladders. The full-length blot was cut and stained with either monoclonal anti-HMGB1, poloyclonal anti-HMGB1, or polyclonal anti-RAGE Ab. (b) All three blots were imaged together at various exposures, including auto, 10, 30, 60 and 120 seconds. (c) Images of blots merged with ladder are shown. esRAGE (51kDa) or rHMGB1 (31kDa) was indicated in a red box.
